# Community health worker training to reduce mental health and substance use stigma towards patients who have disengaged from HIV/TB care in South Africa: protocol for a stepped wedge hybrid type II pilot implementation trial

**DOI:** 10.1186/s43058-023-00537-w

**Published:** 2024-01-02

**Authors:** Bronwyn Myers, Kristen S. Regenauer, Alexandra Rose, Kim Johnson, Sibabalwe Ndamase, Nonceba Ciya, Imani Brown, John Joska, Ingrid V. Bassett, Jennifer M. Belus, Tianzhou (Charles) Ma, Goodman Sibeko, Jessica F. Magidson

**Affiliations:** 1 Curtin enAble Institute, Faculty of Health Sciences, Curtin University, Kent Street, Perth, WA Australia; 2 Mental Health, Alcohol, Substance Use and Tobacco Research Unit, South African Medical Research Council, Parow, Cape Town, South Africa; 3 Department of Psychiatry and Mental Health, Division of Addiction Psychiatry, University of Cape Town, Cape Town, South Africa; 4 Department of Psychology, University of Maryland, College Park, College Park, MD USA; 5 Department of Psychiatry and Mental Health, Division of Neuropsychiatry, University of Cape Town, HIV Mental Health Research Unit, Cape Town, South Africa; 6 Division of Infectious Diseases, Medical Practice Evaluation Center, Massachusetts General Hospital/Harvard Medical School, Boston, MA USA; 7 Department of Clinical Research, Division of Clinical Epidemiology, University Hospital Basel, University of Basel, Basel, Switzerland; 8 Department of Medicine, Swiss Tropical and Public Health Institute, Allschwil, Switzerland; 9 Department of Epidemiology and Biostatistics, University of Maryland, College Park, MD USA

**Keywords:** Stigma, Depression, Task-sharing, Low- and middle-income country, Global mental health

## Abstract

**Background:**

South Africa has deployed community health workers (CHWs) to support individuals to enter and stay in HIV/TB care. Although CHWs routinely encounter patients with mental health (particularly depression) and substance use (SU) conditions that impact their engagement in HIV/TB care, CHWs are rarely trained in how to work with these patients. This contributes to mental health and SU stigma among CHWs, a known barrier to patient engagement in care. Mental health and SU training interventions could reduce CHW stigma and potentially improve patient engagement in care, but evidence of the feasibility, acceptability, and preliminary effectiveness of these interventions is scarce. Therefore, we designed a hybrid type 2 effectiveness-implementation pilot trial to evaluate the implementation and preliminary effectiveness of a CHW training intervention for reducing depression and SU stigma in the Western Cape, South Africa.

**Methods:**

This stepped wedge pilot trial will engage CHWs from six primary care clinics offering HIV/TB care. Clinics will be block randomized into three-step cohorts that receive the intervention at varying time points. The Siyakhana intervention involves 3 days of training in depression and SU focused on psychoeducation, evidence-based skills for working with patients, and self-care strategies for promoting CHW wellness. The implementation strategy involves social contact with people with lived experience of depression/SU during training (via patient videos and a peer trainer) and clinical supervision to support CHWs to practice new skills. Both implementation outcomes (acceptability, feasibility, fidelity) and preliminary effectiveness of the intervention on CHW stigma will be assessed using mixed methods at 3- and 6-month follow-up assessments.

**Discussion:**

This trial will advance knowledge of the feasibility, acceptability, and preliminary effectiveness of a CHW training for reducing depression and SU stigma towards patients with HIV and/or TB. Study findings will inform a larger implementation trial to evaluate the longer-term implementation and effectiveness of this intervention for reducing CHW stigma towards patients with depression and SU and improving patient engagement in HIV/TB care.

**Trial registration:**

ClinicalTrials.gov NCT05282173. Registered on 7 March 2022.

**Supplementary Information:**

The online version contains supplementary material available at 10.1186/s43058-023-00537-w.

Contributions to the literature

- Provider-level mental health and substance use stigma reduction interventions have been tested in low- and middle-income countries but have not been routinely implemented.
- This study will test the implementation of the *Siyakhana* intervention, which targets depression and substance use stigma among community health workers (CHWs) supporting patients with HIV/TB care re-engagement.
- The study will provide some of the first evidence of the feasibility and acceptability of the *Siyakhana* intervention when implemented in South African health services as well as the implementation strategies designed to overcome potential barriers to implementation.
- The study will also provide initial evidence of the effectiveness of this intervention for shifting CHW stigma towards depression and substance use.
- The potential for scalability of the *Siyakhana* intervention is enhanced through the study’s use of a low-cost, existing infrastructure of CHWs, currently deployed throughout South Africa to support patients with TB/HIV care engagement.

## Introduction

Despite South Africa’s efforts to expand HIV and TB treatment, the country continues to struggle with a high incidence of HIV and TB [1]. Challenges with engaging and retaining patients in care contribute to HIV and TB incidence in this setting [1–4]. Evidence suggests that only half of all patients complete TB treatment and less than two-thirds achieve virological suppression on antiretroviral therapy (ART) for HIV [5, 6]. Without implementing strategies to enhance patient engagement in care, South Africa is unlikely to meet global targets for ending HIV and TB by 2030.

In response to this challenge, the South African Department of Health has implemented community health worker (CHW) programs to support TB and HIV care [7, 8]. CHWs conduct home visits to promote care re-engagement for individuals who have fallen out of HIV and/or TB care [8, 9]. During these visits, CHWs often encounter patients with symptoms of SU and depression due to the high prevalence of depression and substance use (SU) in these patient groups [10–12] and the contribution of these symptoms to HIV/TB care engagement difficulties [11–13]. Based on our previous work [14, 15], approximately one-third of patients with HIV/TB may screen positive for SU use or depression symptoms, with higher rates among patients with HIV/TB care disengagement.

Yet, CHWs receive limited training on how to identify and communicate with patients who have symptoms of SU or depression [16, 17]. While some mental health and SU trainings have been piloted for CHWs [16, 18, 19], this training is not consistently provided as part of the Department of Health’s CHW accreditation program. Given the well-documented association between poor mental health literacy and stigma [20, 21], this lack of training likely contributes to CHW stigma towards patients with HIV/TB and depression or SU. Indeed, our earlier work documented high levels of depression and SU stigma among South African CHWs [22–24]. This is cause for concern as mental health and SU stigma among health providers is known to impact healthcare quality, including the implementation of evidence-based practices and patient-centered care [25–27]. In addition, CHW stigma towards people with symptoms of depression or SU is a known barrier to patient re-engagement in HIV/TB care [22, 28, 29]. Consequently, interventions to reduce CHW stigma towards patients with SU or depression symptoms are needed to improve patient engagement in HIV/TB care.

## Study objectives

In response to these knowledge gaps, this study aims to evaluate the initial implementation and preliminary effectiveness of a CHW training intervention for reducing SU and depression stigma towards patients who have fallen out of HIV/TB care in the Western Cape, South Africa.

The primary objective of this study is to evaluate the feasibility, acceptability, and fidelity of implementing this training. A secondary objective is to assess the preliminary effectiveness of this training intervention (compared to usual care) for reducing CHW stigma towards depression and SU towards patients who have fallen out of HIV/TB care. Implementation and preliminary effectiveness outcomes will be assessed at 3- and 6-month post-training timepoints.

## Methods

This protocol is reported in accordance with the guidelines presented in the Standard Protocol Items: Recommendations for Interventional Trials (SPIRIT) checklist [30] and the Consort Guidelines for Reporting Stepped Wedge-Cluster Randomised Controlled Trials [31]. See Supplementary files 1 and 2 for relevant checklists.

### Study design and setting

This is a hybrid Type 2, stepped-wedge cluster randomized controlled pilot trial with a dual focus on the preliminary effectiveness of the training intervention when delivered in a real-world context and initial implementation outcomes, allowing for piloting of implementation strategies [32].

This study is being conducted within the City of Cape Town’s community health clinics located in the Khayelitsha health district within the Western Cape, South Africa. These clinics offer publicly funded, integrated HIV and TB treatment to patients from predominately low-income communities where there is a high prevalence of HIV, TB, SU, and mental health concerns [33, 34]. At these clinics, there is an existing infrastructure of CHWs who provide basic health services and home visits to patients with HIV/TB. The Western Cape Department of Health contracts non-governmental organizations (NGOs) to employ CHWs.

**Fig. 1:**
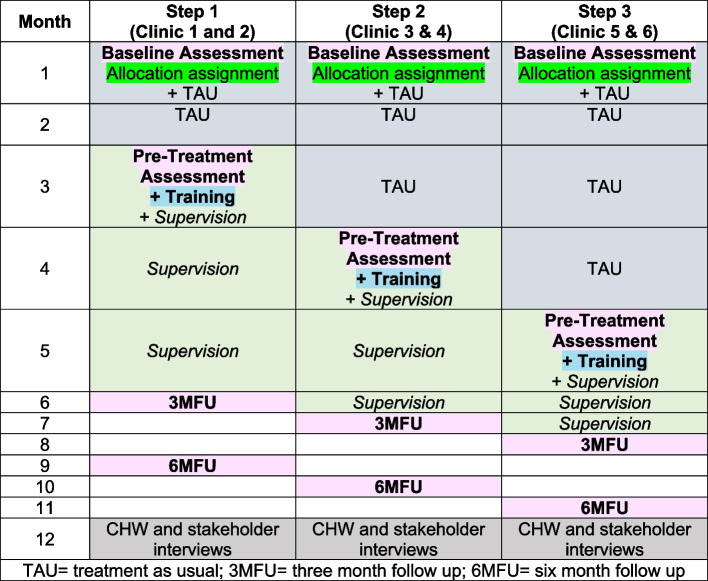
Overview of stepped wedge trial design and timeline. TAU, treatment as usual; 3MFU, 3-month follow-up; 6MFU, 6-month follow-up

In collaboration with the City of Cape Town, we identified six clinics whose CHWs will receive the training. We selected clinics based on their location, the number of CHWs providing HIV/TB care, and NGO support for training of the CHWs that they employ. Each clinic will be randomized to the timing of when their CHWs receive the training intervention (versus treatment as usual) over a 1.5-month period. Clinics will be block randomized into three-step cohorts by the study statistician. Figure 1 presents an overview of the project over the trial period. CHWs within each clinic site who agree to participate will receive the training at their clinic’s assigned time. CHWs are allocated to a single clinic and community, minimizing the potential for contamination. Randomization will not affect any other TB/HIV-related service or mental health training offered to CHWs.

### Participants and procedures

CHWs across all six clinics will be recruited at approximately the same time. We anticipate enrolling a minimum sample of 60 CHWs (10 CHWs per clinic). All CHWs at these clinics will be invited to participate in the training. CHWs will be eligible for study participation if they are at least 18 years old, employed as a CHW through one of two partner NGOs that provide HIV/TB services, and work with patients with HIV/TB who may have depression or substance use concerns. CHWs will be excluded if they are unable to complete the training in English or unable to provide informed consent for study participation or complete study assessments in English or isiXhosa (the main languages of the district). Trained study staff will meet with CHWs to explain the training and study procedures, enquire about their interest in study participation, and conduct eligibility screening. CHWs who are eligible and interested in participating will be asked to complete informed consent procedures followed by a baseline assessment, including self-report questions on demographic characteristics; the CHW role; knowledge of HIV, TB, depression, and SU; and stigma.

In stepped-wedge trials, all groups have different intervals of a baseline period in which they are not exposed to an intervention (which we refer to as “treatment as usual” or “TAU”) [35]. After the baseline assessment, all clusters will be informed of the timing of their training (their allocation). Each cluster will receive at least one dose of TAU. Based on the training time to which their cluster was randomized, CHWs will then be invited to participate in the *Siyakhana* training. In Step 1, clinics randomized to Training Time #1 will receive the training while the other clinics continue to receive TAU. In Step 2, clinics randomized to Training Time #2 will receive the training, while the final set of clinics continue to receive TAU. In Step 3, clinics randomized to Training Time #3 will receive the training.

Before their assigned training, CHWs will complete a pre-training assessment comprising most of the self-report questionnaires asked at baseline. At the pre-training assessment and on the final day of training, CHWs will complete a brief vignette-based role-play of an interaction between the CHW and a hypothetical patient with HIV and depression or SU, played by a staff member who is minimally involved in delivering the training. This will be conducted in the language CHWs use with most of their patients (English or isiXhosa), videotaped, and later coded for the presence of stigmatizing language. All CHW participants will be invited to complete a follow-up assessment approximately 3 months after their training (3MFU) and again approximately 6 months after their training (6MFU), consisting of the same questionnaires from the pre-training assessment, another videotaped roleplay, and questions about implementation outcomes. All assessments and roleplays will be conducted in English or isiXhosa (the main languages of the setting).

After the 6MFU, we will contact a subset of CHWs (up to *n* = 20) who participated in the training for a qualitative interview about their experiences of applying the training content in their interactions with patients and the perceived feasibility and acceptability of the training and their implementation of the training content. For these interviews, we will randomly select CHWs from the following subgroups: CHWs who report a significant decrease in depression/SU stigma, those who report no change in depression/SU stigma, and CHWs who report a significant increase in depression/SU stigma. We will also conduct qualitative interviews (up to *n* = 28) with leadership staff from participating clinics and NGOs and with policymakers and other stakeholders. These interviews will explore their perceptions of the feasibility and acceptability of the training and organizational barriers and facilitators to training sustainment. Interviews will continue until we reach theoretical saturation.

### Interventions

#### Treatment as usual (TAU)

In the TAU period, CHWs will continue to deliver their usual services to patients with HIV/TB who have disengaged from care. These CHWs will have completed standard CHW training provided through the Western Cape Department of Health as part of their employment conditions. This intensive training spans 6 weeks with booster training provided on an annual basis. Training focuses on basic communication skills; information about HIV, TB, and sexually transmitted infections; clinical guidelines for these conditions; training in HIV testing and counseling; and TB/HIV adherence counseling. Adherence counseling focuses on identifying and addressing structural barriers to care engagement [15, 16]. Mental health and SU training is not currently offered as part of CHW certification [16, 17].

#### Siyakhana training

Within a week of completing the pre-training assessment, CHWs will be offered the *Siyakhana* training. The *Siyakhana* training was informed by Link and Phelan’s [36] stigma framework, combined with the Situated Information Motivation Behavioral Skills Model of Care Initiation and Maintenance framework (sIMB-CIM) [37] and adapted based on prior formative work [23, 24], prior training programs to improve CHW mental health and SU literacy and teach problem-solving skills [15, 18, 19, 23]. According to the Link and Phelan framework, mental health, and SU stigma occurs when CHWs label their patients who have SU and/or depression symptoms as different, attach negative stereotypes to them, and distinguish these patients from themselves and their other patients. We included information that challenged CHWs’ preconceptions of patients with SU or depression, including opportunities for direct and indirect contact with people with lived mental health and SU experience, videos of patients talking about their lived experience with mental health and SU challenges and training on non-judgmental communication. Further, the training will be conducted by registered psychological counselors combined with individuals with lived experience of depression/SU.

The sIMB-CIM model recognizes that engagement and retention in health services require accurate information about health conditions, motivation, and skills for overcoming barriers to care. Consequently, *Siyakhana* is designed to provide CHWs with accurate information on depression andSU and evidence-based communication, motivational and problem-solving skills to support non-stigmatizing interactions with patients that may help promote patient re-engagement in care. The *Siyakhana* training will also teach CHWs self-care strategies (see Table 1 for an overview of the training content).

**Table 1:** Siyakhana training components

**Theoretical framework**	• Link and Phelan’s stigma framework informed the design of the stigma reduction component [36]. The Situated Information Motivation Behavioral Skills Model of Care Initiation and Maintenance framework [37] informed training components focused on patient re-engagement in care
**Trainers**	• A psychological counselor, registered with the Health Professions Council of South Africa (HPCSA) with 5 years of experience in motivational interviewing and problem-solving therapy and training CHWs • A peer interventionist
**Training overview**	• 3 days of training covering psychoeducation on depression, SU, stigma, HIV, TB; self-care strategies for CHWs; evidence-based strategies for working with patients who may have depression or SU and patient videos of lived experience with mental health and SU challenges • Mixture of didactic teaching and experiential group activities including skills rehearsal exercises and role plays
**Training content**	
***Day #1***	• Welcome to training, training expectations • *Psychoeducation* on the role of a CHW, depression, substance use, HIV, TB, stigma, and culture ▪ Video shown: patient talking about lived depression experience ▪ Video shown: patient talking about lived substance use experience • *Self-Care Skill*: Mindfulness exercise • *Evidence-Based Skill*: Problem-Solving Skill
***Day #2***	• Welcome, reflection on previous day, reflection on mindfulness • *Evidence-Based Skill for Non-stigmatizing interactions*: Confidentiality, Motivational Interviewing, Nonjudgmental Communication • *Self-Care Skill*: Identifying Values • Challenging situations: brainstorming difficult situations that may arise with patients and/or colleagues
***Day #3***	• Welcome and reflection • Challenging situation: Roleplaying • *Self-Care Skill*: Balancing Values in Life • The value of supervision • Roleplay and rehearsal • Summary and reflection
**Supervisor characteristics**	• Psychological counselor, registered with the Health Professions Council of South Africa (HPCSA) or Social Worker registered with the South African Council for Social Service Professions (SACSSP) • Conduct guided by the professional standards and ethics of the HPCSA/SACSSP
**Supervision structure**	• In-person, group supervision • Up to an hour and a half in duration • Structured to include: *Debriefing, Challenges* to applying training content during patient-interactions, *Refresher training:* Brief skills rehearsal exercises or role playing to refresh training content and consolidate skills

Training will be delivered in English like other CHW trainings offered by the Western Cape Department of Health. Our training team consists of English and isiXhosa mother-tongue speakers and will be able to address questions and further explain concepts in isiXhosa if needed.

### Implementation strategy

The implementation strategy involves two components. First, as intervention components that involve social contact with individuals with lived experience have the most empirical support for stigma reduction [26, 38–41], peers with lived personal and family experience of mental health and SU will help deliver the training. Second, we will provide CHWs with clinical supervision and support to apply the training content in their work with patients. Our formative and prior work with CHW-delivered depression and SU interventions emphasized the importance of supervision to support CHWs to use new skills and to prevent burnout among these frontline workers who witness the difficult circumstances of their patients [16, 42, 43]. Each CHW cluster will receive group supervision. Like previous studies [14, 15, 44], a registered psychological counselor or social worker will provide supervision. During supervision, CHWs will re-cap the skills learned during training, discuss challenges with applying these skills, and receive clinical debriefing and psychosocial support for challenging patient interactions, and for practicing self-care strategies. This supervisor will be supported by psychologists on the research team.

### Study measures

The data collection schedule is shown in Table 2. As this is a hybrid pilot trial, we will assess both implementation and effectiveness outcomes.

**Table 2:** Description of measures and assessment schedule

**Domain**	**Measure**	**Assessment timepoint**				
		***Baseline***	***Pre-training***	***Post-training***	***3 months***	***6 months***
***Implementation outcomes:***						
Feasibility	• CHW attendance in the training (> 75% as benchmark)			X		
	• Feasibility subscale of the Dissemination and Implementation measure [45]				X	X
	• Qualitative interviews					X
Acceptability	• Acceptability subscale of the Dissemination and Implementation measure [45]				X	X
	• Qualitative interviews					X
Fidelity	• Fidelity rating of CHW roleplays (using Fidelity checklist)				X	X
	• Qualitative interviews (barriers to fidelity)					X
***Preliminary effectiveness outcomes:***						
CHW stigma	• Social distance scale [46]	X	X		X	X
	• Coding of stigmatizing responses in role plays based on case vignettes		X		X	X
***Covariates***						
CHW characteristics	• Demographic characteristics, prior mental health and SU training, years in current role, qualifications	X				
History of mental health or SU	• Personal or family history of mental health or substance use concerns	X				

#### Implementation outcomes

Guided by Proctor’s model of implementation outcomes [47], we will use mixed methods to assess the feasibility, acceptability, and fidelity of the CHW training from the perspective of the CHW, organization, and other key stakeholders. Haroz et al.’s [45] dissemination and implementation measure will quantitatively assess implementation outcomes among CHWs at 3MFU and 6MFU. For this measure, items within each of the acceptability and feasibility subscales are rated on a four-point scale (0 = “not at all”, 3 = “a lot”), summed, and averaged for a final subscale score. Feasibility will also be assessed via the percentage of training sessions attended across all CHWs (> 75% of CHW attendance will be used as a benchmark).

We will randomly select 20% of the CHW roleplays conducted at the 3MFU and 6MFU assessments to rate fidelity to the use of a nonjudgmental, non-stigmatizing way of working with patients. Raters will use a pre-determined checklist to assess roleplays for the presence of stigmatizing language and behaviors, delivery of skills learned in training, provision of correct information, and the presence of positive, supportive interactions with patients. A fidelity score will be calculated for each rated roleplay based on the proportion of key intervention components delivered as intended across all interactions. A final fidelity score, made up of the average of individual fidelity scores, will be calculated.

In addition, qualitative feedback from CHWs, organizational leadership, and other key stakeholders will be used to assess feasibility and acceptability of training as well as barriers and facilitators to implementation and fidelity at 6MFU. Interview guides will be developed using open-ended questions and structured probes based on Proctor’s definitions of acceptability, feasibility, and fidelity [47] and the Consolidated Framework for Implementation Research [48].

#### Preliminary effectiveness outcomes

##### CHW stigma

CHW stigma towards depression and SU will be assessed at each timepoint through the Social Distance Scale (SDS), a widely used measure of mental health and substance use stigma [46, 49, 50]. Participants will be presented with a vignette of a patient with HIV and symptoms of depression and another vignette of a patient with HIV and symptoms of a substance use disorder (as defined by the DSM-5). These vignettes were informed by previous work in this setting [51, 52] and our pilot test [24]. Slightly different versions of the vignettes will be presented at each timepoint. For each vignette, participants will be asked to rate their willingness to engage with the hypothetical patient across six social interactions on a 4-point scale from (1) “definitely” to (4) “definitely not”. Scores are summed to create an overall score, with higher scores indicating a desire for greater social distance from the patient, and therefore, greater stigma. To supplement the SDS, CHW roleplays at pre-training, 3MFU and 6MFU will be qualitatively coded for the presence of stigmatizing language and behaviors.

#### Additional measures

CHW and other stakeholder participants will be asked to complete a demographic questionnaire that includes questions about age, gender, education level, primary language, job title, and length of time in their current position and occupation.

### Planned analyses

#### Implementation outcomes (primary analysis)

Quantitative data on feasibility, acceptability, and fidelity will be analyzed descriptively (*M, SD*, range). Thematic analysis [53] will be used to analyze qualitative data on implementation outcomes and barriers. Initial concepts will be identified based on the interview guide and an observation record. Concepts will be used to develop a codebook, and two trained independent coders will code each transcript. NVivo will be used to organize the data according to each code. The research team will review the coded transcripts to determine emerging themes. Final themes will be agreed upon and inter-rater reliability of coding assessed. For the component of this aim that focuses on barriers to implementation, the CFIR [48] will guide coding so that we can identify implementation barriers that require prioritization when scaling the training.

We will integrate qualitative and quantitative data using a convergent, mixed methods design [54]. We will compare qualitative and quantitative data for each implementation outcome separately with an equal emphasis on the qualitative and quantitative. We will create study diagrams for each implementation outcome with the key findings across qualitative and quantitative data in separate columns, allowing for a comparison and integration of findings. We will focus on points for integration where the qualitative and quantitative findings are consistent with each other, contradictory, and/or can provide additional meaning.

#### Preliminary effectiveness outcomes (secondary analysis)

The primary goal of this study is to establish the feasibility and acceptability of the training, and therefore all effectiveness analyses are considered preliminary. We will conduct descriptive analyses (e.g., mean, standard deviation) to summarize CHW stigma, using the continuous SDS score. To account for the stepped wedge study design, we will fit a linear mixed model for depression and a separate model for SU stigma. This analytic approach will allow us to account for both random effects of the clinic and CHW and fixed effects of time and intervention. All models will follow intent-to-treat principles: participants will be analyzed based on their initial randomization assignments and all data will be included, regardless of subsequent dropout or missing data. We will analyze the within- and between-group effects. The within-group effect will evaluate changes in stigma before and after receiving the training with CHWs from the same clinic, and the between-group effect will compare the CHW training to TAU. An F-test will examine the between- and within-clinic intervention effects. Covariates (i.e., CHW demographic and job-related characteristics) will be included in the models. The models will account for any baseline differences in stigma between clinics and individual CHWs.

For these analyses (assuming an intra-class correlation of 0.1 within clinic and 0.3 within person), a minimum of 60 CHWs will need to be enrolled to achieve 86% power at a significance level of 0.05 and a CHW training effect of 0.63, and 96% power to detect a training effect of 0.75. Anticipated standardized effect sizes were defined from a meta-analysis of 13 randomized clinical trials of mental health stigma reduction interventions that show a standardized effect size of 0.63 on measures of stigma [38] and prior work in sub-Saharan Africa using the SDS to test changes in mental health stigma among community mental health volunteers that had an effect size of 0.75 [49].

### Ethics and dissemination

Ethics approval has been granted by the SAMRC’s Human Research Ethics Committee (EC039-10/2021) and the City of Cape Town. The trial is registered on clinicialtrials.gov (NCT05282173). Informed consent will be obtained from all potential CHW and stakeholder participants prior to enrolment. During the consent process, participants will be informed of all foreseeable risks of study involvement, that participation is voluntary, and that they may withdraw their consent without any consequences to their role or employment.

To ensure confidentiality, a unique participant identification number will be used to identify and link the various study forms. We will enter quantitative data into a password-protected REDCap electronic database. These data will be de-identified to protect participants’ identities. All data not stored electronically will be stored in double-locked filing cabinets in designated locked offices. Forms with personal identifying information will be stored separately from case report forms. Video and audio-recordings, identified only by the participant identification number, will be uploaded into a secure database that only study staff can access. Once uploaded, these recordings will be deleted from the recording device.

The main risk associated with this study is the risk of psychological distress or discomfort. To minimize this risk, we will train all research staff to recognize and respond appropriately to signs of discomfort and distress. This will include debriefing with participants and referring them to local mental health and SU services. Our study team includes registered psychological counselors and psychologists who have the correct competencies to debrief distressed participants and ensure they access appropriate care.

Study findings will be disseminated through presentations at local, national and international conferences and peer-reviewed journals. We will also present the study findings to the City of Cape Town and NGOs participating in this implementation pilot trial.

### Data and trial management

A data monitoring committee was not established as this trial involves training health providers in how to work in non-stigmatizing, person-centered ways with patients. Any serious adverse events will be reported to the ethics committee and study sponsor.

### Role of trial sponsor and funder

This pilot implementation trial was funded by the National Institute of Mental Health (NIMH) grant R34MH122268, awarded to Drs. Magidson and Myers. The South African Medical Research Council is as the trial sponsor. The sponsors and funders play no role in study design, data collection, management, analysis, and interpretation of data; writing of the report; and the decision to submit the report for publication.,

## Discussion

Although CHW stigma towards patients with mental health and SU concerns is a major barrier to TB/HIV engagement in low and middle-income countries like South Africa [22, 28], relatively few CHW-level mental health and SU stigma reduction interventions have been tested and implemented in these settings [39–41, 55, 56]. Limited evidence of the feasibility, acceptability, and effectiveness of these interventions for shifting CHW-level stigma has likely affected health service planners' willingness to invest in these interventions.

This hybrid pilot trial will respond to these knowledge gaps by evaluating both the implementation and preliminary effectiveness of a stigma reduction training for reducing CHW stigma towards depression and SU to ultimately improve patient re-engagement in TB/HIV care. Study findings will advance scientific knowledge by providing some of the first evidence for the feasibility, acceptability, and barriers to implementing provider-level stigma reduction interventions within real-world health services. It will also provide some of the first initial evidence of whether this intervention holds promise for shifting CHW stigma towards patients with depression or SU who have disengaged from HIV/TB care.

This trial has several strengths. First, the *Siyakhana* training is innovative as it includes both education and contact-based components. Previous studies from LMICs have not evaluated the effects of combining these evidence-based intervention components on provider stigma towards people with mental health and/or SU concerns [56]. Second, in contrast to other provider-level stigma reduction interventions that target only one stigmatized condition, this intervention targets both depression and SU stigma. If we find promising intervention effects for both types of stigmas, this will suggest that the *Siyakhana* training could offer a wider range of benefits than a training focused on mental health or SU stigma alone. This could increase the chances of future implementation in resource-constrained health services. Third, this study will assess whether changes in stigma are evident at 6MFU, adding to the very limited evidence of the sustained effects of these interventions [41, 56]. Fourth, this study’s testing of the feasibility and acceptability of an intervention and its implementation strategy addresses a critical gap in the mental health and SU stigma reduction literature from LMICs [55]. Fifth, this study will leverage a low-cost, existing infrastructure of CHWs, currently deployed throughout South Africa to trace patients with TB/HIV who have disengaged from care in their homes; maximizing opportunities for wider adoption and scale up of this intervention should the findings from future implementation of the training in a fully powered implementation trial suggest benefits for patient engagement in care.

We have anticipated potential challenges to study implementation. First, CHW turnover is high in this setting and may impact on CHW retention in the study at 6MFU. To limit participant attrition, we will employ similar retention strategies to those we have used in other CHW trials where we have obtained 6MFU rates greater than 80% [15]. Second, there may be other initiatives to support CHWs to deliver mental health and SU interventions to people with HIV/TB that may affect trial findings. To account for this, we will monitor and document any additional training that CHWs are exposed to during the study.

In summary, this study will yield important information on the feasibility and acceptability of the *Siyakhana* intervention and its implementation strategy as well as preliminary information on the effectiveness of this training for reducing CHW stigma towards depression and SU. Should this pilot study yield promising implementation and effectiveness outcomes, findings will be used to inform a larger trial to evaluate the longer-term implementation and effectiveness of this intervention for reducing CHW stigma towards patients with depression and SU concerns and improving patient engagement in HIV/TB care.

## Trial status

CHW and stakeholder recruitment commenced on 8 June 2022 and is expected to conclude in January 2024.

### Supplementary Information

**Additional file 1.** SPIRIT 2013: Recommended items to address in a clinical trial protocol.

**Additional file 2.** Consort checklist for stepped wedge trials.

## Data Availability

De-identified data and study materials are available upon written request.
